# Contribution of Conventional Cardiovascular Risk Factors to Brain White Matter Hyperintensities

**DOI:** 10.1161/JAHA.123.030676

**Published:** 2023-07-08

**Authors:** Fatemeh Koohi, Eric L. Harshfield, Hugh S. Markus

**Affiliations:** ^1^ Stroke Research Group Department of Clinical Neurosciences University of Cambridge Cambridge United Kingdom

**Keywords:** cardiovascular risk factors, magnetic resonance imaging, structural equation modeling, white matter hyperintensities, Magnetic Resonance Imaging (MRI), Vascular Disease, Risk Factors, Cardiovascular Disease

## Abstract

**Background:**

White matter hyperintensities (WMHs) are a major risk factor for stroke and dementia, but their pathogenesis is incompletely understood. It has been debated how much risk is accounted for by conventional cardiovascular risk factors (CVRFs), and this has major implications as to how effective a preventative strategy targeting these risk factors will be.

**Methods and Results:**

We included 41 626 UK Biobank participants (47.2% men), with a mean age of 55 years (SD, 7.5 years), who underwent brain magnetic resonance imaging at the first imaging assessment beginning in 2014. The relationships among CVRFs, cardiovascular conditions, and WMH volume as a percentage of total brain volume were examined using correlations and structural equation models. Only 32% of the variance in WMH volume was explained by measures of CVRFs, sex, and age, of which age accounted for 16%. CVRFs combined accounted for ≈15% of the variance. However, a large portion of the variance (well over 60%) remains unexplained. Of the individual CVRFs, blood pressure parameters together accounted for ≈10.5% of the total variance (diagnosis of hypertension, 4.4%; systolic blood pressure, 4.4%; and diastolic blood pressure, 1.7%). The variance explained by most individual CVRFs declined with age.

**Conclusions:**

Our findings suggest the presence of other vascular and nonvascular factors underlying the development of WMHs. Although they emphasize the importance of modification of conventional CVRFs, particularly hypertension, they highlight the need to better understand risk factors underlying the considerable unexplained variance in WMHs if we are to develop better preventative approaches.

**Nonstandard Abbreviations and Acronyms**
CVRF: cardiovascular risk factor
CVC: cardiovascular condition
SEM: structural equation modeling
SVD: small‐vessel disease
UKB: UK Biobank
WMH: white matter hyperintensity

**Clinical Perspective**

**What Is New?**

- This is the largest study to date assessing the proportion of white matter hyperintensity (WMH) risk accounted for by conventional cardiovascular risk factors.
- We found that all common conventional cardiovascular risk factors combined explained only 15% of the variance in WMHs, highlighting the limited explanatory power of cardiovascular risk factors alone in understanding the development of WMHs.

**What Are the Clinical Implications?**

- Despite the importance of conventional cardiovascular risk factors, other vascular and nonvascular factors are involved in the development of WMHs.
- Further research is needed to identify and understand additional factors underlying the considerable unexplained variance in WMHs, which may provide insights into novel targets for intervention and prevention strategies for WMHs.

Cerebral small‐vessel disease (SVD) is a major global cause of stroke and dementia.^1^ White matter hyperintensities (WMHs) are a key magnetic resonance imaging (MRI) marker of SVD, and they have been shown to predict both stroke and dementia.^2^ Increasingly, WMHs are being used to monitor SVD progression and as a surrogate disease marker for clinical trials in SVD.^3^, ^4^

Currently, there are few proven treatments for SVD, and better understanding of the underlying disease mechanisms has been highlighted as important in developing better treatment approaches.^5^ One approach has been to investigate and target risk factors for WMHs. Epidemiologic studies suggest a familial component,^6^ and recent genome‐wide association studies have identified multiple genetic loci associated with WMH risk.^7^, ^8^ Cardiovascular risk factors, particularly hypertension, have also been implicated as risk factors for WMHs.^9^, ^10^, ^11^

However, there has been uncertainty about the proportion of WMH risk that is accounted for by conventional cardiovascular risk factors (CVRFs). A recent study suggested that only 2% of the total risk could be accounted for by all common vascular risk factors.^12^ This finding was perhaps unexpected in view of the many previously reported associations between conventional CVRFs and WMHs but has major implications for the proportion of WMH risk that could be targeted by risk factor control. In addition, it is unclear whether vascular risk factors are independently associated with increased WMHs, or whether there are common underlying factors that influence both vascular risk factors and the presence of WMHs.^13^

Therefore, using the large and well‐characterized population of the UK Biobank (UKB), we sought to determine the proportion of variance in WMHs accounted for by conventional CVRFs.

## Methods

The UKB data that support the findings of this study are publicly available to bona fide researchers on application at http://www.ukbiobank.ac.uk/using‐the‐resource/.

### Study Population

The UKB is a large, prospective, population‐based cohort study that recruited >500 000 community‐dwelling participants, aged 40 to 69 years, across Great Britain between 2006 and 2010. The UKB study design and population have been described in more detail elsewhere.^14^ Following the initial assessment, starting in 2014, a subset of 100 000 participants began undergoing brain MRI.^15^ In this study, we included all 42 940 UKB participants who had undergone brain MRI at the first imaging assessment.

### Standard Protocol Approvals, Registration, and Patient Consents

UKB received ethical approval from the National Information Governance Board for Health and Social Care and the National Health Service Northwest Multicenter research ethics committee. All participants provided informed consent through electronic signature. The present analyses were conducted under UKB application number 36509.

### Measures of CVRFs and Conditions

CVRFs were assessed at baseline recruitment for each participant at a UKB assessment center via a touchscreen questionnaire and physical measurements.

Weight was measured using the Tanita BC‐418MA body composition analyzer (Tanita Corp, Tokyo, Japan). Height was measured using the Saca 202 device in a barefoot standing position. Body mass index was derived as weight in kilograms divided by height in meters squared. Waist circumference was measured with a Wessex nonstretchable sprung tape measure (Andover, UK).

Systolic blood pressure and diastolic blood pressure were taken as the average of 2 measurements in the sitting position after a 5‐minute rest using an Omron 705IT digital monitor. Hypertension was defined as systolic blood pressure ≥140 mm Hg, diastolic blood pressure ≥90 mm Hg, or taking blood pressure medications.

Glycated hemoglobin was measured by high‐performance liquid chromatography analysis on a Bio‐Rad VARIANT II Turbo. Diabetes was defined on the basis of elevated levels of glycated hemoglobin, taking high blood glucose medications, self‐reported data, interviews, or hospital inpatient records. Cholesterol, high‐density lipoprotein cholesterol, low‐density lipoprotein cholesterol, and triglycerides were measured by direct enzymatic methods. Hyperlipidemia was defined as elevated levels of total cholesterol (≥240 mL/dL), low‐density lipoprotein cholesterol (≥160 mg/dL), or triglycerides (≥200 mg/dL), or low levels of high‐density lipoprotein cholesterol (<40 mg/dL).

Cardiovascular conditions (CVCs) were defined for a history of clinical outcomes (namely, stroke, coronary artery disease, and myocardial infarction) and clinical risk factors (ie, atrial fibrillation), using algorithmically defined outcomes, from hospital admission records, self‐report at nurse interview, or death certificate records, which were recorded before the date of imaging assessment.

### Measures of WMHs

We used image‐derived variables provided by the UKB team for total WMH volume (using T1‐ and T2‐weighted fluid‐attenuated inversion recovery images) and total brain volume (derived as the sum of white matter volume and gray matter volume from T1 images, normalized for head size, and measured in cubic millimeters).^15^

The details of the MRI acquisition protocol and pipeline for the production of imaging‐derived phenotypes have been described elsewhere.^16^ Briefly, all brain MRI data were acquired on a single standard Siemens Skyra 3T scanner with 32‐channel head coils. To transform the original T1‐ and T2‐weighted fluid‐attenuated inversion recovery images into MNI152 space, spatial normalization procedures were performed on these images. After gradient distortion correction and reduction of the field of view to remove nonbrain tissue, a nonlinear registration to 1‐mm resolution MNI152 space was done using the functional magnetic resonance imaging of the brain (FNIRT) nonlinear image registration tool. All of the above transformations estimated are then combined into 1 single nonlinear and reversible transformation.^16^

WMHs were automatically segmented using the Brain Intensity Abnormality Classification Algorithm tool^17^ and the combined T1‐ and T2‐weighted fluid‐attenuated inversion recovery data as input. Brain Intensity Abnormality Classification Algorithm is an automated supervised method for WMH segmentation based on the k‐nearest neighbor algorithm and voxel intensity. The total WMH volume was calculated from the voxels inside a white matter mask that had a probability of being WMH >0.9.^17^

We calculated WMH percentage volume by dividing total WMH volume by total brain volume and applied a log transformation to approximate a normal distribution.

### Statistical Analysis

Descriptive statistics were presented as means (SDs) for continuous data and frequencies (percentages) for categorical data. Continuous variables with highly skewed distributions, such as triglycerides, were log transformed.

We imputed missing data for CVRFs and CVCs based on chained equation methods (10 imputations) using the mice (3.15.0) package.^18^ Among the included participants with complete data on WMH volume, the proportion of originally missing values that were substituted with values obtained by multiple imputation ranged from 0.0% to 8.8% per variable (Table 1).

**Table 1:** Baseline and WMH Characteristics of Participants Based on Both Nonimputed and Imputed Data

Characteristics	Missing data	Nonimputed data	Imputed data
No. of participants		41 626	41 626
Age, y	0	54.99 (7.53)	54.99 (7.53)
Sex, male	24 (0.06)	19 632 (47.16)	19 649 (47.20)
Cardiovascular risk factors			
Waist circumference, cm	1 (0.002)	87.83 (12.49)	87.83 (12.49)
Body mass index, kg/m^2^	0	26.54 (4.20)	26.54 (4.20)
Systolic blood pressure, mm Hg	5 (0.01)	135.26 (17.85)	135.26 (17.85)
Diastolic blood pressure, mm Hg	5 (0.01)	81.29 (9.91)	81.3 (9.9)
Blood HbA1c, mmol/mol	2485 (5.97)	35.01 (5.09)	35.0 (5.1)
Triglycerides, mmol/L	2281 (5.48)	1.64 (0.96)	1.64 (0.95)
Hypertension	15 (0.04)	19 270 (46.29)	19 273 (46.30)
Diabetes	12 (0.03)	2185 (5.25)	2185 (5.25)
Hyperlipidemia	3680 (8.84)	20 115 (48.32)	21 133 (50.77)
Cardiovascular conditions			
Stroke	0	557 (1.34)	557 (1.34)
Coronary artery disease	1369 (3.29)	2326 (5.59)	2335 (5.61)
Myocardial infarction	0	894 (2.15)	894 (2.15)
Atrial fibrillation	1362 (3.27)	1849 (3.27)	1881 (4.52)
WMH volume, %	0	0.35 (0.47)	0.35 (0.47)

Data represent mean (SD) for continuous variables and frequency (percentage) for categorical variables. HbA1c indicates glycated hemoglobin; and WMH, white matter hyperintensity.

We first examined the bivariate associations between CVRFs and WMH volume using Pearson correlations for continuous variables and biserial correlations for dichotomous variables. We excluded risk factors with weak correlations (<0.10), including smoking, alcohol consumption, physical activity, total cholesterol, low‐density lipoprotein cholesterol, high‐density lipoprotein cholesterol, and non–high‐density lipoprotein from the remainder of the analyses.

**Figure 1:**
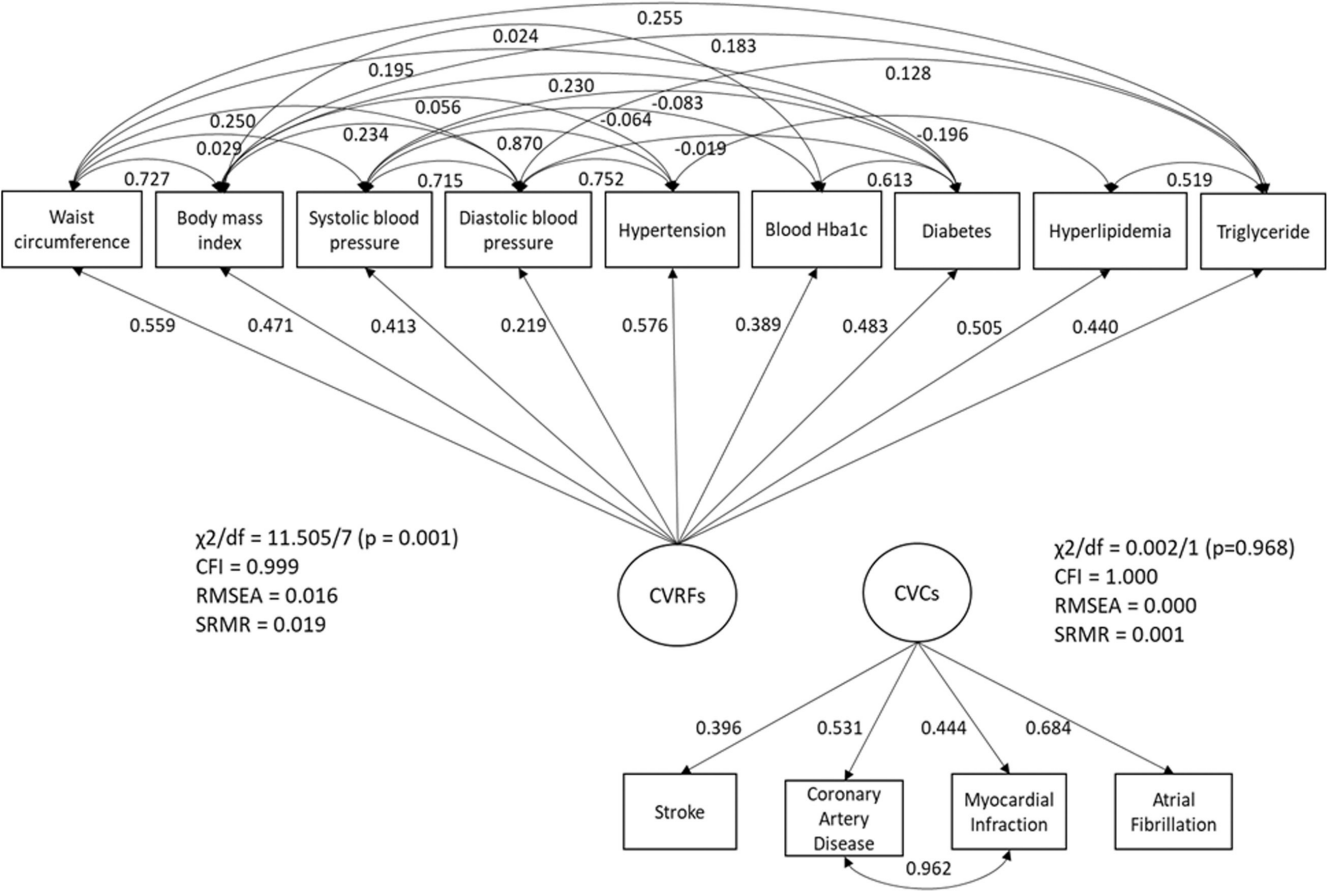
Path diagram of measurement models for the cardiovascular risk factors (CVRFs) and cardiovascular conditions (CVCs) latent constructs. The rectangles denote measured variables, and the circles represent latent variables (CVRFs or CVCs) that are a weighted combination of the measured variables, and they were given labels based on the concept they represent. Single‐headed arrows are hypothesized causal pathways, and double‐headed arrows are correlations; numbers adjacent to the arrows are standardized loading factors and residual correlations, respectively. Model fit parameters are shown adjacent to each measurement model. The models and parameters are estimated by the confirmatory factor analysis. CFI indicates comparative fit index; HbA1c, glycated hemoglobin; RMSEA, root mean square error of approximation; and SRMR, standardized root mean square residual.

**Figure 2:**
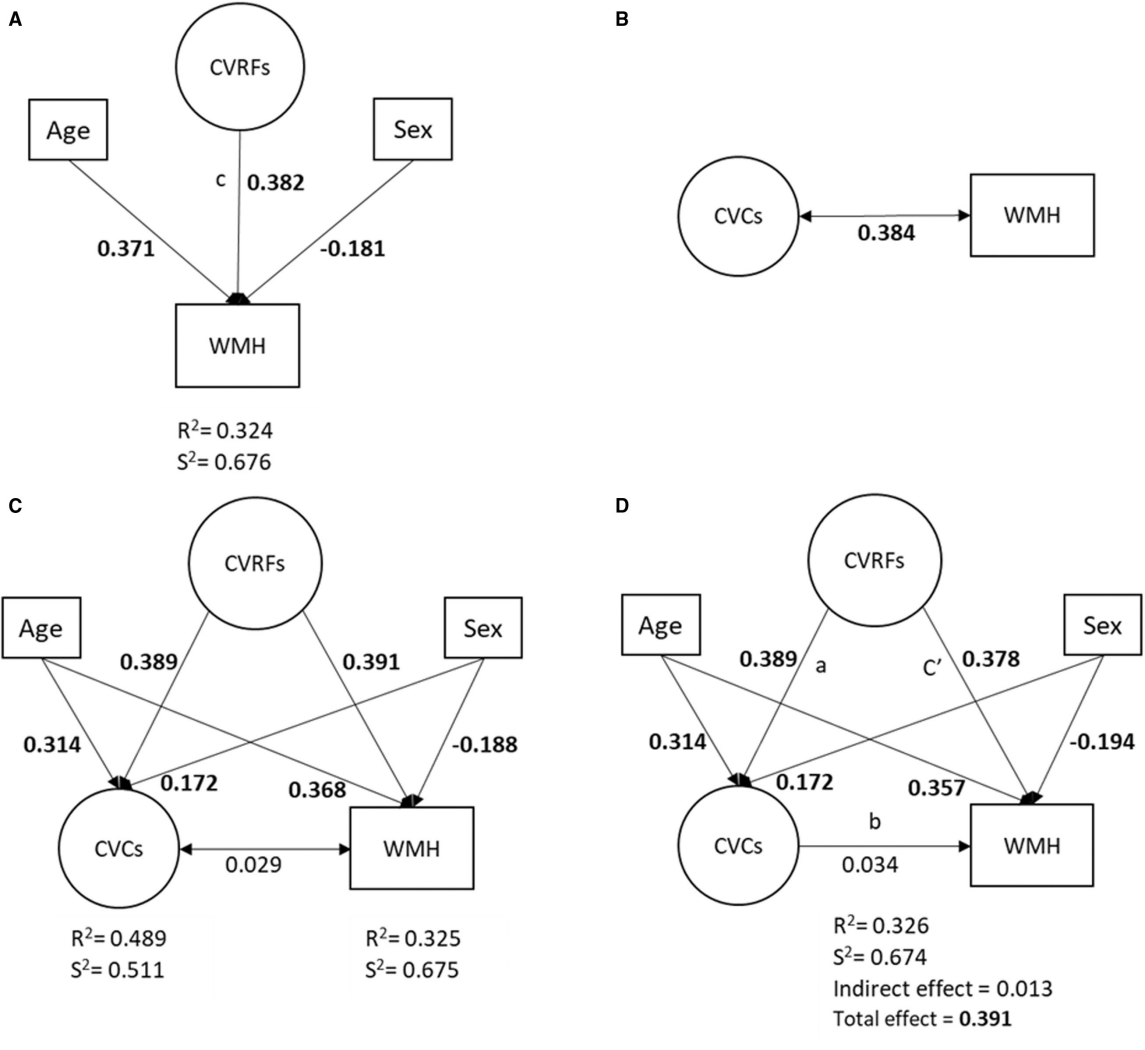
Path diagram of structural models for white matter hyperintensity (WMH) volume. **A**, The total effect of cardiovascular risk factors (CVRFs) on WMH volume. **B**, The undirected association (correlation) between cardiovascular conditions (CVCs) and WMH volume. **C**, The effect of CVRFs on both CVCs and WMH volume. **D**, The direct, indirect, and total effect of CVRFs on WMH volume (the mediation model). **A**, **C**, and **D** were adjusted for sex and age at baseline. Standardized regression coefficients are shown adjacent to each path; significant coefficients are shown in bold. All the models and parameters are estimated by structural equation modeling. *R* ^2^ indicates the variance explained by the model; and *S* ^2^, the residual variance in WMH volume that is unexplained by the model.

We used structural equation modeling (SEM) to assess the magnitude of contributions of CVRFs and CVCs to the WMH variation. SEM, by definition, is a combination of statistical techniques, including multiple regression, confirmatory factor analysis, and path analysis, that allows for the simultaneous estimation of multiple relationships and interrelationships among measured variables and conceptual unmeasured variables (latent variables), considering the interdependencies among them. The purpose of an SEM is to account for variation and covariation of the measured variables. Such models include 2 sets of models: the measurement model and the structural model. The measurement model examines the relationship between the latent variables and their measured variables. The structural model defines the relationship between latent variables and other measured variables that are not indicators of another latent variable. A path diagram is typically used to conceptualize an SEM (eg, Figures 1 and 2), in which the rectangle (or square) boxes represent measured variables, circles represent latent variables that are a weighted combination of the correlated measured variables and are given meaningful labels based on the theoretical meaning or concept they represent, single‐headed arrows denote simple regression relationships, and double‐headed arrows signify correlations.^19^, ^20^

To implement the SEM, we applied a 2‐step modeling approach recommended by Anderson and Gerbing.^21^ The first step involved assessing the measurement models to evaluate the validity of the latent constructs. A confirmatory factor analysis was conducted to test the goodness of fit between the observed indicators and each latent construct. Various fit indexes were examined to evaluate the fit of the measurement models, including comparative fit index, root mean square error of approximation, and standardized root mean square residual. We considered a comparative fit index >0.90, a root mean square error of approximation <0.06, and a standardized root mean square residual <0.08 to be acceptable model fit indexes.^22^, ^23^ In the second step, we computed the structural models based on the measurement models identified in the first step. An SEM was applied to fit the measurement and structural models of regression relationships between the variables simultaneously. The fit of the structural models was assessed using the same fit indexes mentioned in the first‐step analysis. We used the “semTools” package (version 0.5–6)^24^ to fit confirmatory factor analysis and SEM models to multiple imputed data sets.

We describe measurement models for the latent constructs for CVRFs and CVCs in Figure 1. The CVRF latent construct comprised continuous measured variables, including waist circumference, body mass index, systolic blood pressure, diastolic blood pressure, glycated hemoglobin, and triglycerides, and dichotomous history variables, including hypertension, diabetes, and hyperlipidemia. The CVC construct included variables for history of clinical cardiovascular outcomes, consisting of stroke, coronary artery disease, and myocardial infarction, and clinical risk factors comprising atrial fibrillation before the date of imaging assessment. For any 2 variables that were correlated, the covariance between these 2 variables was considered in the model (ie, double‐headed arrows in Figure 1) to correct for an overestimation of the relationship between independent and dependent variables.^20^ Figure 2 shows structural models of relationships among these latent constructs (CVCs and CVRFs) and WMH volume. Model A determined how much variance in WMH volume was attributed to CVRFs. In models B and C, we examined the undirected association between CVCs and WMH volume, and the hypothesis that this association may have arisen because of the presence of CVRFs as a common cause of CVCs and WMHs. Therefore, it would be expected that any association between CVCs and WMH volume (Figure 2B) would diminish after controlling for CVRFs in model C (Figure 2C). In model D, we assessed the hypothesis that the causal relationship between CVRFs and WMH volume (Figure 2A) may be mediated by CVCs (Figure 2D). Age and sex were controlled in models B, C, and D. We estimated model parameters via weighted least squares and assessed the indirect effect in the mediation model (model D) using a Monte Carlo CI derived on the basis of empirical sampling distributions of estimated model parameters.^24^, ^25^, ^26^

Finally, we investigated which of the CVRFs considered in the primary analyses accounted for most of the variation in WMH volume. We also explored how the contribution of each individual risk factor to WMH volume varied by age categories.

All analyses were performed using R version 4.1.1 (R Core Team, 2021). A 2‐sided *P*<0.05 was considered statistically significant.

## Results

A total of 41 626 of the 42 940 UKB participants who had undergone brain MRI at the first imaging assessment had data on WMH volume. Table 1 provides sample characteristics for these participants, based on both nonimputed and imputed data. Mean age was 55 years (SD, 7.5 years), and 19 632 (47.2%) were men. Approximately 46% of the participants had hypertension, 5% had diabetes, and 48% had hyperlipidemia at baseline. About 1% had a stroke, 5.6% had coronary artery disease, 2% had myocardial infarction, and 3% had atrial fibrillation before the date of imaging assessment.

The measurement models for deriving the CVRFs and CVCs latent constructs with their standardized loading factors and residual correlations are presented in Figure 1. All observed variables were significantly associated with their respective latent constructs (*P*<0.05). Because of the large sample size, significant χ^2^ tests were obtained for the CVRF model; however, other model fit statistics indicated good model fit (Figure 1). Figure 2 depicts the structural models and the standardized estimates for WMH volume. Model fit statistics indicated good fit for all structural models.

### CVRFs and WMH Volume

After adjustment for age and sex, the standardized total effect of CVRFs explained ≈15% of the variation in WMH volume (standardized β=0.382; Figure 2A). The *R* ^2^ value indicated that only 32% of the variance in WMH volume was captured in history measures of CVRFs, sex, and age. Age was by far the strongest predictor of WMH, accounting for ≈16% of the variation in WMH volume.

**Figure 3:**
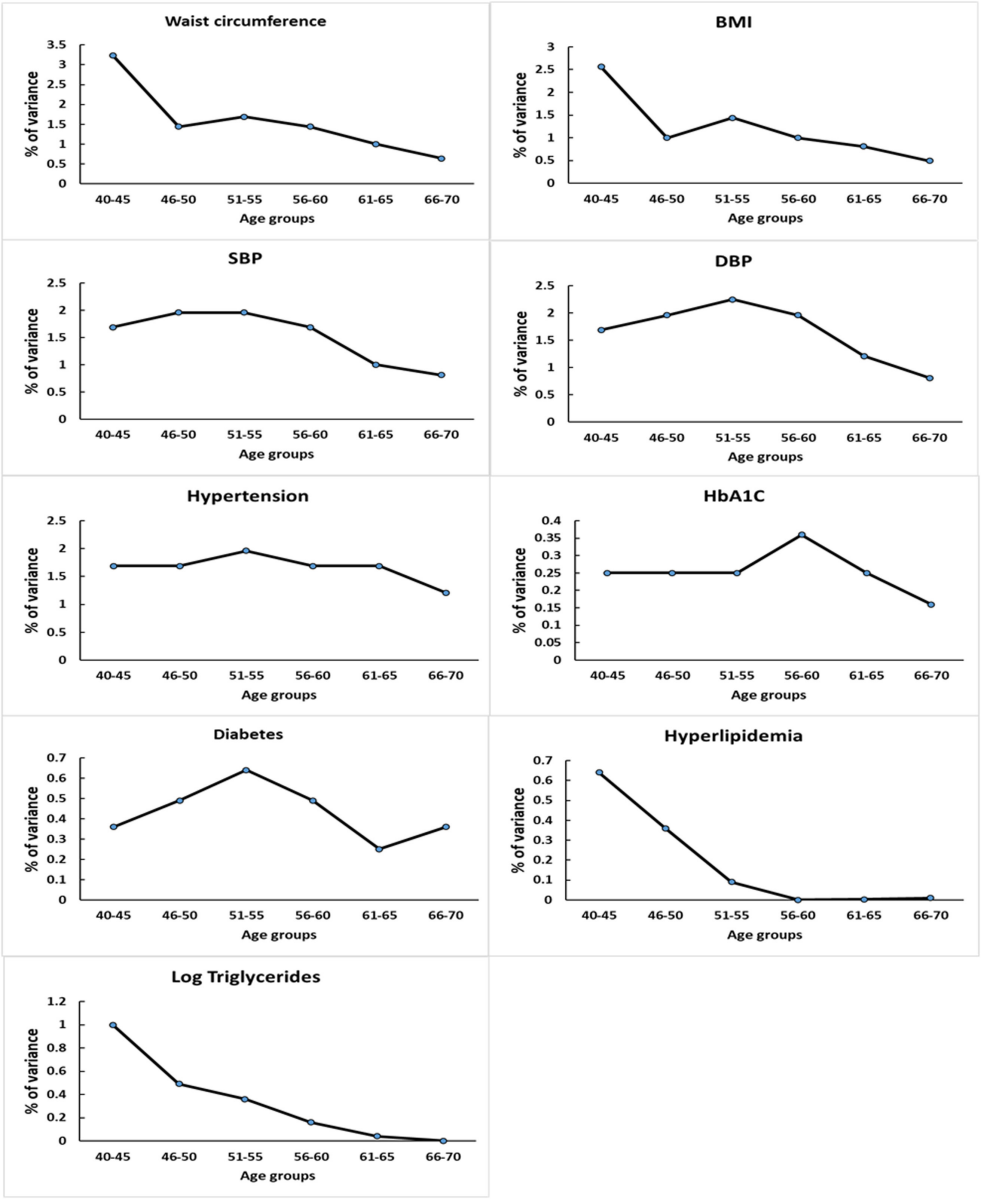
Patterns of change in the shared variance between individual risk factors and white matter hyperintensity volume, calculated by multiplying the squared correlation coefficients by 100, according to age categories. BMI indicates body mass index; DBP, diastolic blood pressure; HbA1c, glycated hemoglobin; and SBP, systolic blood pressure.

The contribution of individual risk factors to WMH risk is shown in Table 2. The strongest risk factors were blood pressure and hypertension, with the blood pressure parameters together accounting for ≈10.5% of total variance in WMH (diagnosis of hypertension, 4.4%; systolic blood pressure, 4.4%; and diastolic blood pressure, 1.7%). Moreover, as age increased, the amount of shared variance between most of the individual risk factors and WMH volume decreased, indicating that the contribution of CVRFs to WMH declined with age (Figure 3).

**Table 2:** Amount of Shared Variance Between Each CVRF and WMH Volume

CVRF	WMH volume	
	Correlation coefficient	% of variance*
Waist circumference	0.14	2.0
BMI	0.09	0.8
SBP	0.21	4.4
DBP	0.13	1.7
Hypertension	0.21	4.4
HbA1c	0.12	1.4
Diabetes	0.08	0.6
Hyperlipidemia	0.07	0.5
Triglycerides	0.07	0.5
Sex	0.08	0.6
Age	0.37	13.7

BMI indicates body mass index; CVRF, cardiovascular risk factor; DBP, diastolic blood pressure; HbA1c, glycated hemoglobin; SBP, systolic blood pressure; and WMH, white matter hyperintensity.

* The percentage of shared variance, calculated by multiplying the squared correlation coefficients by 100.

### Relationship Between WMH Volume and CVCs

There was a shared variance of ≈15% between CVCs and WMH volume without accounting for CVRFs (Figure 2B). However, this undirected association disappeared when the CVRFs were accounted for in model C (Figure 2C), indicating that CVRFs increased both CVCs and WMH, and that the association between WMH and CVCs is largely explained by the association between CVRFs and WMH. In addition, CVRFs were a similar predictor of both WMH volume and CVCs, accounting for ≈15% of variation in each of them (standardized β=0.391 for WMH percentage and 0.389 for CVCs).

In mediation analyses that considered CVCs as a mediator on the causal pathway between CVRFs and WMH measures, with adjustment for age and sex, there was no significant indirect effect of CVRFs on WMH volume through CVCs, and the standardized total effect of CVRFs was the same as for models A and C (Figure 2D). This is consistent with WMHs increasing cardiovascular outcomes largely via their association with CVRFs.

## Discussion

In a large community cohort of >40 000 participants, we found that common CVRFs combined accounted for ≈15% of the variation in WMH volume, with age accounting for an additional 16%. However, a large portion of the variance, and well over 60%, remains unexplained.

Previous studies have reported a wide variation in the importance of CVRFs in WMH risk. Indeed, it has been suggested that they may play only a minor role. One recent study of older subjects from 2 independent cohorts, one community dwelling and the other with stroke, found that all common vascular risk factors in both populations explained only 2% of the variance in WMHs.^12^ Our findings, in a larger sample size than previous studies, confirm they do play a significant role, even if much variance remains unexplained. However, the blood pressure–related variables accounted for over half the total variance explained by CVRFs, and of the other risk factors, only waist circumference (2%) and diabetes‐related variables (history of diabetes, 0.6%; and glycated hemoglobin, 1.4%) each accounted for 2% of the total variance. We also found that the shared variance between most risk factors and WMH volume decreased with age. This is consistent with previous research^27^, ^28^ and emphasizes the importance of targeting interventions for modifiable cardiovascular risk factors from midlife to prevent both WMHs and the clinical consequences of SVD.

Our results have direct implications for which treatments may delay WMH progression. The predominant contribution of hypertension is consistent with previous reports,^24^ and suggests that modification of this risk factor is likely to have the greatest effect on reducing WMH in the population, and possibly therefore its related complications of dementia and stroke. This is consistent with the results of the recent SPRINT (Systolic Blood Pressure Intervention Trial), in which intensive blood pressure lowering reduced the progression of WMHs,^29^ and reduced incidence of both stroke^30^ and the combined end point of mild cognitive impairment and dementia.^4^ Of the other CVRFs, only improved diabetes control and anti‐obesity measures, which will also improve diabetes control, are likely to have significant population effects on reducing WMHs. Our results are consistent with Mendelian randomization studies using data from large‐scale genome‐wide association studies, which showed associations of genetically elevated systolic blood pressure, diastolic blood pressure, and body mass index with WMHs, but there was not enough evidence for associations with total cholesterol, low‐density lipoprotein cholesterol, smoking, and type 2 diabetes.^11^

We also explored the relationship between WMHs, CVRFs, and CVCs. Several studies have previously shown that atrial fibrillation,^31^, ^32^ heart failure,^33^, ^34^ and coronary artery disease^35^ are associated with WMHs. In the previous study by Wardlaw et al, they did not find large‐artery atheromatous disease to be directly associated with WMHs.^12^ Likewise, in the current study, we did not find a direct association between the considered CVCs and WMH volume. Instead, the findings of our study indicated that CVRFs, acting as shared contributors to disease in both the heart and the brain, mainly explain any apparent association between CVCs and WMHs. Furthermore, our mediation analysis revealed that the effect of CVRFs on the risk of WMHs was not mediated by the CVCs. This highlights the presence of other strong mechanisms that explain the missing variance in WMHs, emphasizing the heterogeneous cause of WMH development already suggested in previous research.^36^

Over half the risk of WMH was unexplained by CVRFs and age, suggesting other strong, yet unidentified, risk factors. Both family history studies^6^ and more recent genome‐wide association studies have suggested genetic factors are important.^8^ Recent genome‐wide association studies have identified a large number of genes affecting diverse pathways implicated in WMH risk; such genes seem to both predispose to CVRFs, such as hypertension, and act independently by increasing vascular injury and brain responses to injury.^7^, ^8^ Several other disease mechanisms have been proposed,^37^ including inflammation^38^ and blood‐brain barrier permeability.^37^ Better understanding the risk factors that account for the considerable unexplained variance in WMH risk is a priority to allow better preventive and treatment strategies.

Our study has several strengths. It included a large sample size, considerably larger than previous studies, including >41 000 community‐dwelling individuals. MRIs were performed with a uniform high‐quality image acquisition protocol in the UKB with automated WMH delineation.

However, our study also has limitations. A community population sample recruited by random sampling selective response, as in the UKB, has resulted in participants being generally healthier than the overall population, which might limit the generalizability of the results. Another limitation of our study could be that as we used single time point measures of variables, the direction of the associations could not be assessed to infer causality, which is a particular note in the context of SEM, where temporality cannot be assessed at a single time point. Future studies could examine these relationships longitudinally. An additional limitation could be that we used WMHs estimated by the UKB pipeline, in which the size of the ventricles, which is known to increase with age, was not explicitly accounted for and so may affect the segmentation of periventricular WMHs in older individuals. However, the UKB pipeline incorporates several quality control measures to ensure the accuracy of normalization processes, such as excluding individuals with large structural deviations.^16^ Finally, we were unable to include additional imaging markers of SVD, such as lacunar infarcts, cerebral microbleeds, and perivascular spaces, as this information was not made available in UKB at the time of analysis.

In conclusion, we showed that 15% of the risk of increased WMH could be accounted for by conventional cardiovascular risk factors and that blood pressure parameters were the strongest single cardiovascular risk factors for WMHs. However, much of the variance remains unexplained, suggesting other underlying vascular and nonvascular factors that may represent potential therapeutic targets.

## Sources of Funding

This research was funded by a British Heart Foundation program grant (RG/F/22/110052). Infrastructural support was provided the Cambridge British Heart Foundation Centre of Research Excellence (RE/18/1/34212) and by the Cambridge University Hospitals National Institute for Health and Care Research (NIHR) Biomedical Research Centre (NIHR203312). Eric L. Harshfield is supported by the Alzheimer's Society (AS‐RF‐21‐017). Hugh S. Markus is supported by an NIHR Senior Investigator Award. The views expressed are those of the authors and not necessarily those of the NIHR or the Department of Health and Social Care.

## Disclosures

None.
